# Women in a safe and healthy urban environment: environmental top priorities for the women’s presence in urban public spaces

**DOI:** 10.1186/s12905-023-02281-8

**Published:** 2023-04-06

**Authors:** Ali Reza Sadeghi, Elahe Sadat Mousavi Sarvine Baghi, Fatemeh Shams, Sina Jangjoo

**Affiliations:** 1 Department of Urban Planning and Design, Faculty of Art and Architecture, Shiraz University, Shiraz, Iran; 2 Department of Urban Planning and Design, Faculty of Art and Architecture, Tarbiat Modares University, Tehran, Iran; 3 Askew School of Public Administration and Policy, College of Social Sciences and Public Policy, Florida State University, Tallahassee, FL USA

**Keywords:** Urban public spaces, Women, Environmental priorities, Safety and security, Shiraz

## Abstract

Today, in developing countries, women's need to be present in urban public spaces and the establishment of everyday social interactions seem to be ignored more than other groups. Therefore, the present study aims to achieve the environmental components related to women’s presence in urban spaces and prioritize them. In this study, by scrutinizing the criteria obtained from the interviews, a questionnaire was prepared and randomly distributed among 256 women in the population. IBM SPSS has been used to analyze the data and explain the priorities. The results of the T-test show that security (T-value = 6.508 in T1 test), compatibility with behavioral patterns (T-value = 4.975 in T2 test), eventuality (T-value = 11.064 in T3 test), permeability (T-value = 10.220 in T4 test), attention to climate (T-value = 5.692 in T5 test), liberty (T-value = 11.184 in T6 test), collective memory (T-value = 7.367 in T7 test), variety (T-value = 1.816 in T8 test), complexity (T-value = 13.228 in T9 test), and identity (T-value of 18.905 in T10 test) are the most important criteria in motivating presence in urban public spaces, respectively. According to the results, among the individual characteristics of the respondents, the components of collective memory (*r* = 0.805), identity (*r* = 0.784), liberty (*r* = 0.703), and safety and security (*r* = 0.644) have had a positive correlation with the age of the respondents. The results of this study indicated that improving security (individual, social, and psychological) is the main environmental priority for women over 18 to be present in urban public spaces.

**Supplementary Information:**

The online version contains supplementary material available at 10.1186/s12905-023-02281-8.

## Highlights

• Women’s presence in public spaces is affected by environmental factors of built environment.

• Among environmental priorities affected on women’s presence in public spaces, Security is most significantly factor in motivating women for active social participation in urban public spaces.

• Among the individual characteristics of the respondents, the components of collective memory, identity, freedom, and safety have had a positive correlation with the age of the respondents.

**Supplementary Information:**

The online version contains supplementary material available at 10.1186/s12905-023-02281-8.

## Introduction

Public spaces are places of social interaction that cover groups with different interests and are accessible to all. They play a role in a community's collective identity as they display their users' culture and values. In recent decades, different ways of using urban spaces have been the subject of many studies that are not limited to only anthropology, geography, politics, architecture, and urban planning [22]. According to a general definition in democratic societies, urban public spaces belong to all people to enjoy their nature and shape collective spaces for forming social events [49]. This is while before the gender equality approach in urban space design and planning, they were designed as places for men’s presence. This approach has led to creation of a gendered space that only considers men’s needs in urban spaces [52]. The analyses by women’s rights activists show that the built space and environment affect gender. In general, it can be said that women’s access to different parts of the city, especially public spaces, is more limited compared to men's access [23]. However, the concept of women’s presence as citizens in urban spaces has been accepted in pluralistic societies that believe in equal rights between men and women [52].

Many studies showed how women are present and active in urban spaces and their limitations. According to a research institute in France in 2005, the highest rates of rape and violation of women’s rights occur in urban public spaces [6]. In the studies dealing with this issue, some dependent and independent variables have been mentioned, a summary of which is presented in Table 1.

**Table 1:** Methods, dependent, and independent variables were used in some related studies to women and urban public space

Researcher(s)	Dependent variable	Independent variable	Method
Sadeghi & Jangjoo (2022) [54]	Women’s Presence in Urban Public Spaces	Individual Characteristics: 1. marital status 2. age Physical Characteristics of Built Environment: 1. Accessibility and Permeability 2. Security 3. Land use and Activity 4. Environmental and Visual Comfort 5. Facilities and Services	The methods of this research were descriptive-analytical and survey (Sample size of 100 women). The authors used Cramer's V, Kendall's tau coefficient, and simple linear regression to analyze the significant relationship between the physical variables of the built environment and individual variables and the number of times women are present in urban spaces
Khalili & Nayyeri Fallah (2018) [31]	Role of social indicators on vitality parameter to enhance the quality of women's communal life within an urban public space	Functional Perspective: 1. Accessibility 2.Convenience 3. Space Fluency Visual Perspective: 1. Visibility 2. Legibility 3. Human Scale Social and Cultural Perspective: 1. people presence 2. mix-used functions 3. diversity of activities 4. Privacy 5. Familiarity	Several data collection methods are used: -semi-structured individual interview (*n* = 24) -semi-structured focus group interviews (five groups including 28 middle-aged women -direct observation and taking photo-graphs -unobtrusive behavioral observation
Rayatidamavandi, Faizi, Mozaffar (2016) [50]	Assessing Design Principles of Urban Parks in Iran for Promoting Women’s Satisfaction	1. participatory design 2. designing communal spaces 3. Urban security policies 4. pavement and flooring 5. open space design 6. design with water 7. Access	The data related to 41 indexes, according to the questionnaire, was analyzed by a factor analysis model and finally 13 factors were chosen. In this step of the study, multiple linear regressions were used. a D-W test was used to overcome this issue
Soltani & Ghanbari (2014) [59]	Proper Evaluation of Urban Public Spaces for Women in Kermanshah	1. Safety and Security 2. Vitality and dynamism 3. Access and communications 4. Comfort 5. Events of public spaces	Methodology is based on objective, functional analysis. First through library studies, searching for information in databases and then use the questionnaire. based on Cochran formula, the number of samples is 400 questionnaires and sampling is random
Borumand & Rezaee (2014) [9]	Evaluation the performance of the parks in promoting the gender equality in cities	1. Space performance (Duration of the presence, times of presence, scale of doing social activities 2. Freedom of presence (Type of coverage, ease in behavior, peace, independence) 3. Access (Ease of movement, readability, public transportation to/from the park) 4. Security (Ability to stay, time of presence, incidence of crimes and harassments)	Methodology of the research is evaluative-correlative based on questionnaire Prepared based on the Likert scale, the questionnaire of the research included 30 questions. The results were analyzed using Pearson correlation coefficient in SPSS software and then the Friedman test was used to rank the sub factors. Finally, the factors and sub-factors were prioritized using Analytic Hierarchy Process in Expert Choice software
Kareem& Lwasa (2013) [29]	Gendered Spaces in development cities	1. Sanitation Services (drainage systems, public toilets and waste skips) 2. Urban Transportation 3. Urban Physical Infrastructure (lighting, parking, …) 4. Recreation and Social Amenities	The study uses an end-user service satisfaction survey across the five divisions using an open-ended questionnaire. A purposive sample of 500 respondents was selected, and further sub-divided into 100 respondents per division

It seems that studies on how women are present and active in urban spaces can be categorized into some approaches: gender equity, sociological and civic approach, behavioral approach, and psychological approach. While these studies have addressed the typologies, forms, functions, and dimensions of urban public spaces, they often need a sociological perspective that acknowledges the issue of gendered experience in these studies. Typologies of public spaces have evolved, focusing on changes in people’s lifestyles and attitudes. While politics, paying attention to the environment, economics, and civic culture are widely studied to suggest better urban public spaces, studies, and planning policies related to women and public spaces are still rare. The present study has pointed out the designed theories that deal with urban public spaces in detail, highlighting the women being neglected in constructing these spaces and their uses and sense of belonging. All these factors limit women in public spaces and the essential activities that keep public spaces active and alive. In addition, in the existing theoretical structures, women’s presence in urban spaces has been underestimated or often oversimplified, and minimal effort has been made to identify and analyze the relationship between environmental components in urban spaces and women’s presence in these spaces. Thus, the relationships between the environmental components in urban spaces are still very vague.

On the other hand, there seems to be no consensus on what criteria and perspectives should be adopted for women’s presence in urban public spaces. Every author or policy-maker derives his/her definition based on the specific criteria of the field or the study's perspectives, making it challenging to achieve a general definition. As a result, the literature that focuses on women’s environmental priorities for presence in urban public spaces is limited to the extent that a comprehensive study of this concept has not yet been made. The major gap in these studies is the lack of a comprehensive approach to connecting environmental factors and women’s presence in urban spaces. Thus, the present study seeks to introduce the components related to women’s presence in urban spaces to examine their role and effectiveness, especially the environmental components. Accordingly, the main question proposed in this study is:

Is there any significant relationship between the environmental components in the public spaces of Shiraz and women’s presence in such spaces?

In this regard, the research hypothesis states that there is a significant correlation between environmental and physical components in the city and women’s presence, and by strengthening some of these environmental components, women’s presence in urban spaces can be expected to increase.

## Literature review

### The concept of presence of urban spaces (presence of citizens in urban public spaces)

A successful urban space is a liveable, sociable, and highly frequented space with the qualities of attractiveness [11], animatedness, meaning a place for the constant moving of people, accessibility, comfortableness, liveliness, and security [36]. The presence of people in urban spaces is accompanied by safety and social security issues. The more the natural presence of people decreases, the greater the risks will be [10]. However, many studies have pointed to people’s fear of crime in public spaces and have shown that a high percentage of people’s fear of urban public spaces stems from their gender. Even if the probability of crime is generally higher in men than in women, women feel more insecurity and fear in public spaces [44]. Therefore, urban designers and planners always look for solutions to ensure women’s security in public spaces by considering behavioral and psycho-social characteristics and cultural changes. Women’s security is influenced by urban design choices, the organization of urban services, and the integration of urban functions ([60]: 304–305). Additionally, Sadeghi et al. [55] indicate that the structural characteristics of the urban environment have a significant relationship with the subjective well-being of the people living there.

Undoubtedly, various personal and environmental factors influence women’s behavior in urban spaces. Prior studies have mainly studied this subject from the perspective of individual characteristics and the social environment in which a person lives. In this research, the physical environment has been examined as an influencing factor, and its indicators have been introduced.

#### Individual factors

According to the World Health Organization’s (WHO) definition, individual factors are introduced as internal factors that include gender, age, coping styles, social background, education, profession, past and current experience, overall behavior pattern, character, and other indicators that affect a person's abilities. Individual factors are a symbol of individual life and include characteristics that are not mainly related to physical health but have a positive or negative effect on individual performance [21]. To be more precise, a person's behavior is influenced by personal factors, and personal factors also affect their behavior by influencing a person's decisions [72].

#### Environmental factors

Environmental factors affect the quality of life and urban sustainability and are a multifaceted concept resulting from aggregating qualities from different areas [42]. According to prior studies, environmental factors can be studied in two categories of environmental indicators, such as air pollution and noise pollution, as well as spatial quality indicators. In the current study, environmental factors mean the environmental indicators affecting the quality of the space, which are mentioned in the Figure below.

### Gendered urban open spaces and women’s presence in urban public spaces

The Behavioural, environmental, perceptional, structural, and social aspects significantly influence women’s social interactions [58]. In recent decades, many studies have addressed gender as influencing people’s desire to be present in public spaces [38, 61]. Studies show that, on average, the willingness to be present in urban spaces for recreation is less in women than in men [61]. Gender is one of the most frequently studied demographic variables affecting the fear of crime in urban public spaces. There are differences in how women and men perceive security [46]. Generally, any activity in space can be affected by three types of constraints that differ in gender groups [37]. These constraints limit activity spaces among men and women because they specify what activities are allowed at what time and place (e.g., having access to a specific area by women) [37]. According to Hirdman (1990) [26], the position of women in society is determined by men. These men give them less space and freedom of action and movement, limiting women’s activities [8].

Therefore, today achieving a kind of collective space that can establish a sustainable balance between the presence of different social classes and their participation in urban public spaces. In the meantime, some criteria seem to affect women’s presence in urban public spaces.

**Fig. 1:**
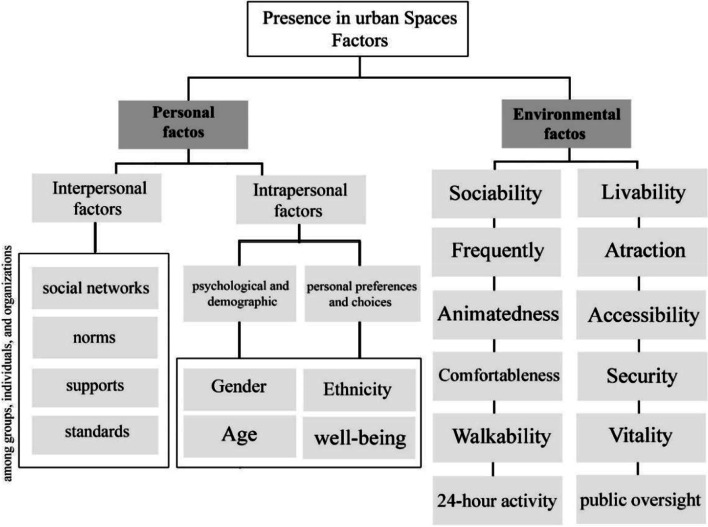
Components related to presence in urban spaces

**Fig. 2:**
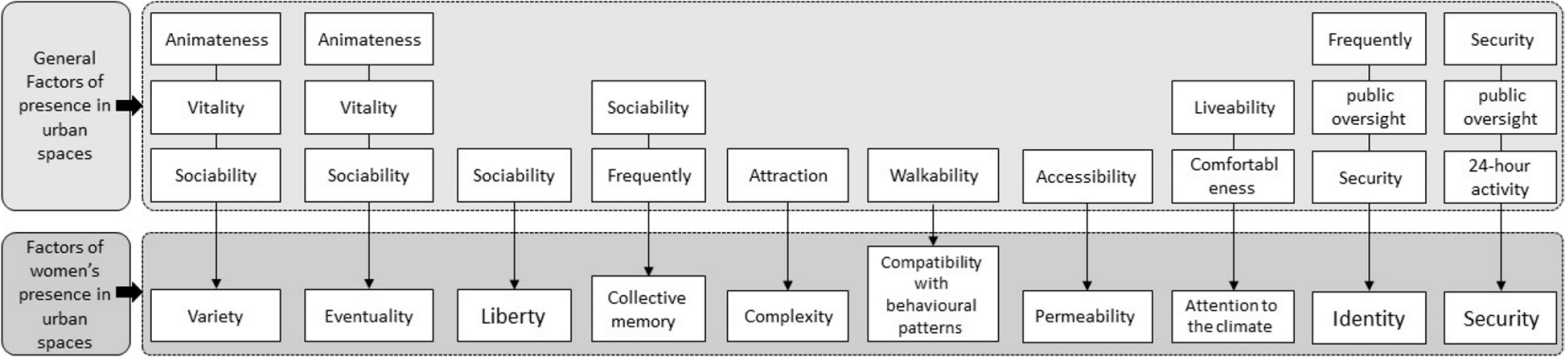
The relationship between general environmental factors of presence in urban spaces and environmental factors encouraging the presence of women in urban spaces

Environmental factors affecting the presence of people in urban spaces were introduced in general (Fig. 1). Then, according to the gender characteristics of urban public spaces, environmental factors encouraging the presence of women in urban spaces were presented (Table 2). Figure 2 illustrates a conceptual framework of research and a comparative approach explaining shows the relationship between general environmental factors affecting the presence of people in urban public spaces and environmental factors encouraging the presence of women in public urban spaces.

**Table 2:** Summary of environmental characteristics related to women’s presence in urban public spaces based on theoretical foundations

Environmental priorities	Concept/ Description	References
Security	Safety and security are among the most important qualities of public spaces. Safety refers to rapid protection of the spaces and their users against potential hazards. The ability to observe the activities and functions of semi-public and public spaces creates a sense of comfort in the observer and thus promotes social security of the public space	Sadeghi & Jangjoo (2022) [54]; Arjmand (2016) [3]; Sadeghi, et al. (2016) [53]; Harvey, et al. (2015) [24]; Krenichyn (2004) [33]; Durmisevic & Sariyildiz (2001) [12]; Navarrete-Hernandez et al. (2021) [43]; lak et al. (2020) [35]
Compatibility with behavioral patterns	Optimal distribution of urban uses and activities encourages people to be present in the space and walk between different places. Comfort includes the equipment and spaces related to sitting and waiting activities and other facilities for users of public spaces	Askarizad & Safari (2020) [4]; Khalili & Nayyeri Fallah (2018) [31]; Van Hagen & Bron (2014) [67] Jin & Whitson (2014) [28]; Matsuoka & Kaplan (2008) [40]
Eventuality	Citizens deserve experiencing desirable, lively, and dynamic public spaces. Cleanliness of public spaces and their proper maintenance encourages people to be present in the space, which in turn promotes a sense of security and reduces fear	Fine (2020) [16]; Kern (2016) [30]; Thompson (2002) [64]
Permeability	Permeability includes destination accessibility. Accessibility provides opportunities for citizens to participate in urban activities. If this quality is poor, it will exacerbate socio-economic and health disadvantages	Sadeghi & Jangjoo (2022); Arjmand (2016) [3]; Beebeejaun (2017) [7]; Weber (2003) [69]; Purcell (2003) [48]
Attention to the climate	Climatic comfort and favorable temperature conditions increase the number of people present in the urban space. Many studies have shown that urban green space has a direct relationship with people’s perception of diversity, unity, beauty, and attractiveness of space; and in addition to balancing the temperature and creating a favorable micro-climate, provides the basis for more presence and staying of people in urban spaces	Sadeghi & Jangjoo (2022) [54]; Williams, et al. (2019) [71]; Schofield & Gubbels (2019) [56]; Amindeldar, et al. (2017) [2]; Thorsson et al. (2007) [66]; Thorsson et al. (2004) [65]; Eliasson (2000) [13]
Variety	Variety is a vital quality for urban spaces which is achieved through variety of components that provide freedom of choice and variety of users who have access to all parts of the space, which enhances monitoring of urban spaces. Variety includes land use mix or job housing/population balance	Hataminejad, et al. (2020) [25]; Raymond, et al. (2010) [51]; Ewing & Cervero (2010) [14]; Matsuoka & Kaplan (2008) [40]
Complexity	The amount of visual richness of space is called complexity, which is created through the diversity and number of components existing in the urban space. There is a direct relationship between the complexity and attractiveness components. But the relationship between complexity and desirability is direct before it reaches its peak and then is indirect	Khalili & Nayyeri Fallah (2018) [31]; Maclean (2018) [39]; Ewing & Clemente (2013) [15]; Portella (2007) [47]
Collective memory	A space that has the potential of recalling several events seems more attractive to the audience	Lak & Hakimian (2019) [34]; Bagheri (2014) [5]
Identity	The overall quality of a space can be calculated by measuring the richness of its identity and psychological and sociocultural meanings, as well as the criteria related to physical comfort, safety, and performance. The identity of a space derives from its symbolic meanings, which arise from the memories, ideas, and feelings associated with that space	Golkowska (2017) [20]; Jabareen (2009) [27]; Khan (2007) [32]; Secor (2004) [57]; Williams & Vaske (2003) [70]; Vaske & Kobrin (2001) [70]
Liberty	Urban space is an all-encompassing space in which the general public is free to perform various social activities	Sunikka-Blank, et al. (2019) [63]; Gholamhosseini, et al. (2019) [19]; Sunaryo, et al. (2013) [62]

Table 2 summarizes the studies conducted on the criteria of women’s presence in urban spaces. In this study, these criteria are called environmental priorities because they are characteristics of the environment (here, urban public spaces) that, from the viewpoint of women, their existence is a priority for their presence in the environment, compared to other environmental characteristics*.*

**Fig. 3:**
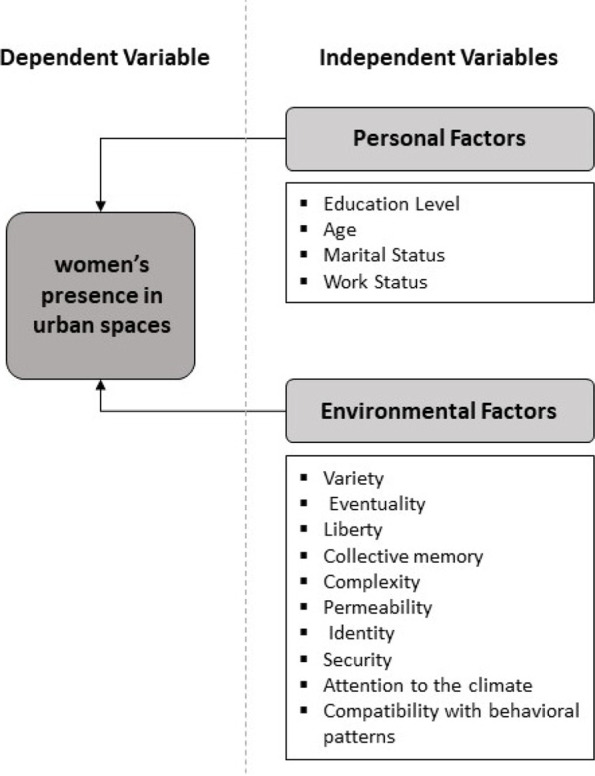
Conceptual framework of research

Using the variables affecting the presence of women in urban spaces that have been introduced in this research, the conceptual framework of the research has been set. According to the studies conducted in this research, the independent variables are divided into personal and environmental factors, and the questionnaire is set based on these variables. Figure 3 shows environmental and personal variables separately.

## Materials and methods

**Fig. 4:**
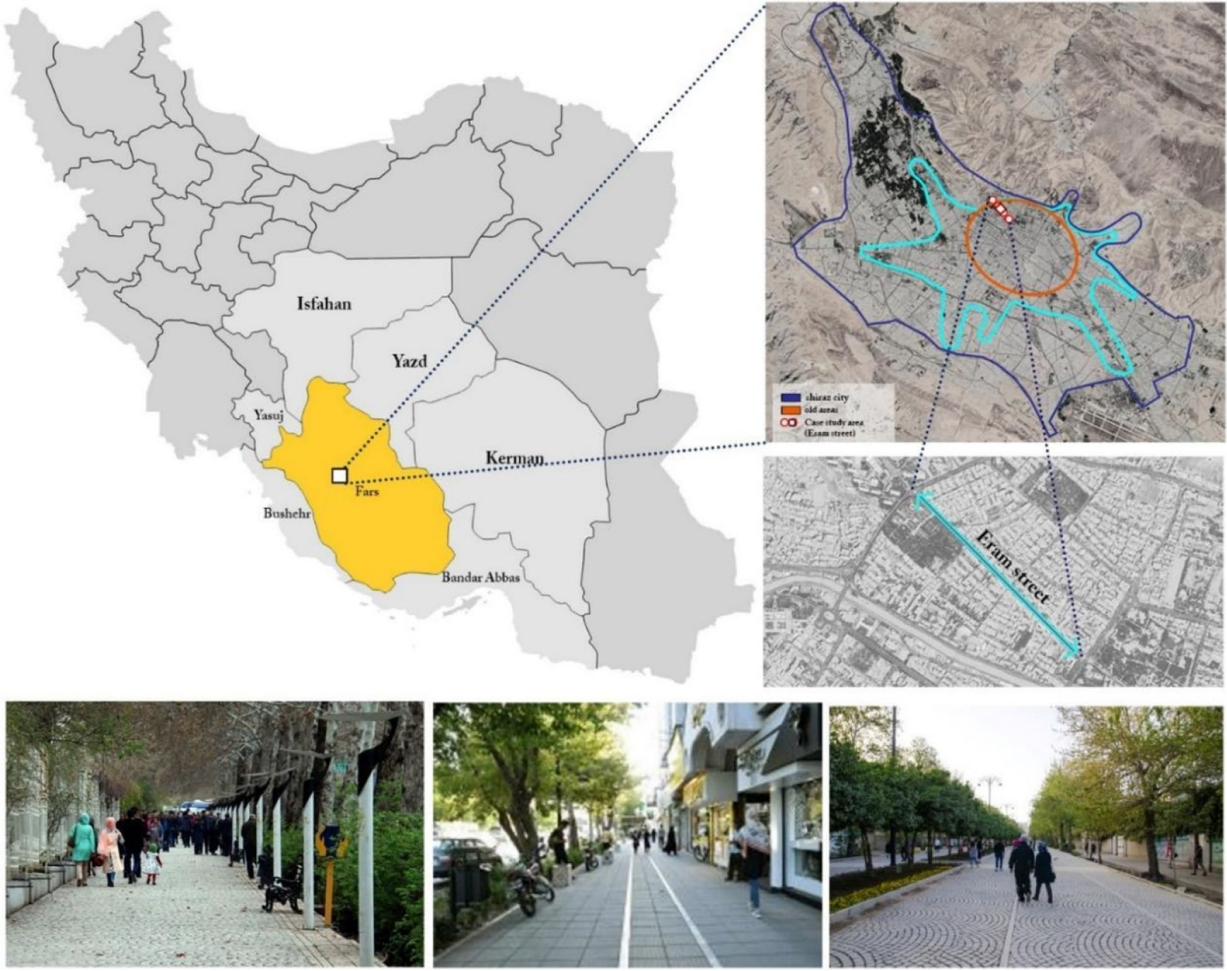
The location of case study (Eram Street) in Shiraz, Iran

The present study aims to prioritize the criteria effective in promoting the over 18 women in Shiraz in urban public spaces to select the main priority from the perspective of this group and also to examine the correlation between the respondents’ personal characteristics and the components related to their willingness to be present in the urban spaces. Shiraz is one of the metropolises of Iran and the capital of Fars province, located in the south of Iran. Based on the last census of Iran in 2016, the population of women in Shiraz was half of the city's total population and was about 780 thousand people. Thus, paying attention to the quality of spaces in a way that provides a favorable environment for the presence of women in urban spaces is crucial. Since the streets play a significant role in the presence of women in urban public spaces and their daily interactions with others, one of the most important streets of Shiraz, named Eram, was selected as a case study. Eram Street is well-known among citizens and even those who travel to Shiraz. Presence in Eram Street is affected by the defined activities around it, such as Eram Garden, restaurants, educational centers, and offices, and different groups are present in this space with different goals, such as leisure and education. Figure 4 illustrates the location of Eram Street in Shiraz*.*

The study is a descriptive-analytical survey. First, to achieve the criteria affecting the presence of this group in urban public spaces, 10 components in the form of 34 questions were set up to conduct interviews with 50 people from the population in Shiraz (as a pilot). These criteria and questions were designed by summarizing the results obtained from the theoretical foundations and conducting in-depth interviews with 14 urban designers and psychologists. It should be noted that interviews have been conducted with women over 18 to ensure that these criteria are compatible with the needs and behavioral patterns of the population.

Therefore, according to the obtained results, 10 criteria of permeability, safety and security, compatibility with behavioral patterns, eventuality, paying attention to climate, complexity, variety, liberty, identity, and collective memory have been introduced as the environmental priorities affecting the presence of the population in urban public spaces of Shiraz. Finally, to determine the importance of environmental priorities, the authors have prepared a questionnaire. This questionnaire consists of two parts: a) demographic information of the respondents (age, education, employment status, and marital status), and b) the appropriateness of the proposed environmental characteristics perceived by the respondents has been evaluated in the form of 34 questions with a 5-point Likert scale (from strongly agree to disagree strongly).

The propositions in the questionnaire are divided into encouraging factors for the presence of women in urban spaces as follows.

Since the content validity method was used to determine the validity and reliability of the research tool, the questionnaire was provided to one of the psychology professors of Shiraz University and after obtaining his comments and doing necessary corrections, the validity of the research tool was confirmed. The Cronbach’s alpha method has been used to determine the reliability of the questionnaire. Since to calculate the Cronbach’s alpha coefficient, the variance of the scores of each subset of questionnaire questions and the total variance should be calculated, 256 questionnaires were distributed among the population and the obtained answers were tested using SPSS software. The coefficient of 0.81 was obtained for all questions. Thus, the questionnaire has good reliability. The population was all women over 18 from Shiraz who use urban public spaces to perform unnecessary activities. Based on the Cochran formula, because the population size was uncertain (assuming that the population is not specified), the sample size in this research was 256. The calculation of the sample size based on the Cochran formula was as follows:

$$n=\frac{{z}^{2}pq}{{e}^{2}}=\frac{{\left(1.96\right)}^{2}(0.5)(0.5)}{{(0.1)}^{2}}=96$$

where “n” is the sample size, “z” is the standard error associated with the chosen level of confidence (typically z = 1.96), “e” is the desired level of precision (e = 0.1), “p” is the estimated proportion of an attribute that is present in the population (*p* = 0.5), and “q” is 1-p (q = 0.5). According to the formula, the minimum acceptable sample size with an acceptable standard error (assuming e = 0.1) is 96. However, due to enhancing the accuracy, more people were studied to ensure acceptable results in this study.

Normality of the data was obtained as 0.067 using the Kolmogorov–Smirnov test. Since its value is greater than 0.05, the data are in the normal range.

Random sampling was used. For 19 days, from the beginning of June to July 2021, researchers were present at Eram Street. They asked women over 18 years who were crossing or doing optional activities in public spaces to fill out the questionnaire (inclusion criteria). For the researchers to have the most contact with the entire studied population, they were in the street on working days and holidays and at different hours of the day and night. By asking some basic questions about whether they were willing to talk about their presence and the characteristics of the environment or not, people who did not want to express their feelings were excluded from the questioning (exclusion criteria). Due to the high number of questions, the effort was to read them to the people so they would be more encouraged to answer. Also, an explanation was given to the person about the purpose of the research.

The analytical statistics to achieve the first goal of the study, namely prioritizing the criteria affecting promoting the women’s presence over 18 in Shiraz in urban spaces, has been provided based on T-test given the normality of the data. For the second goal, namely examining the degree of correlation between individual characteristics of respondents and the components related to their desire to be present in urban spaces, has been provided based on Cramer's V and Pearson correlation tests. Marital status and work status are nominal variables, and education and age are ordinal variables. Therefore, Cramer's V correlation coefficient was used to examine the relationship between marital status and job status and components related to women’s presence in the urban space. Pearson correlation was used to examine the relationship between respondents’ age and education and components related to their desire to be present in the urban space.

## Results

**Fig. 5:**
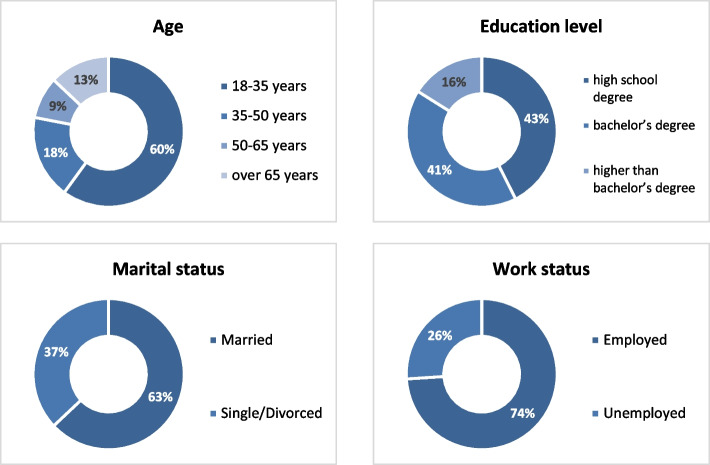
The frequency of demographic variables

Descriptive statistics and demographic examination of the population have been performed using Excel software. The age of individuals has selectively been considered in four categories: a) 18–35, b) 35–50, c) 50–65, and d) over 65. According to the demographic assessments, 60% of the respondents were in the age group A, 18% in the age group B, 9% in the age group C, and 13% in the age group D. 28.9% of the respondents had a high school degree, 28.1% a bachelor’s degree, and 10.9% higher than bachelor’s degree. In addition, 63% of the respondents were married and 74% were employed (Fig. 5).

As mentioned earlier, the main purpose of the study was to determine the degree of importance of each of the main environmental priorities of the population to be present in urban public spaces. Therefore, a T-test has been done among the 10 environmental priorities of the population that have affected the presence of this group in urban public spaces, and the degree of importance of each environmental priority has been determined based on the absolute value of the smallest T value. At each stage of the T-test, if one or more environmental priorities have had equal absolute values, the one with the lowest T value has been selected. In addition, if an environmental priority has been repeated consecutively in the stages of the T-test, the first value obtained has been determined and it has been ignored in the later stages of the T-test. The results of this test are presented in Table 3. According to the T-test results, the environmental priorities related to the women’s presence over 18 in Shiraz are: 1) safety and security, 2) compatibility with behavioral patterns, 3) eventuality, 4) permeability, 5) attention to climate, 6) liberty, 7) collective memory, 8) variety, 9) complexity, and 10) identity, respectively. Table 4

**Table 3:** Statements of the questionnaire

Factors	Items
security	1.The space be of mental health atmosphere
variety	2.People of different ages attend that space
Compatibility with behavioral patterns	3.View, paint, and materials used in the space be in harmony with the surrounding environment
Compatibility with behavioral patterns	4.The place being far or near is of no importance
Compatibility with behavioral patterns	5.The public space be clean and far from sound and air pollution
security	6.The space be defensible (little crime and malefaction be committed in the space)
permeability	7.Public access to the public urban space be considered
variety	8.Diverse uses and performance be available in the space
variety	9.Creative and diverse color composition be used in the space
Compatibility with behavioral patterns	10.There be integrity in the whole space and little dispersion of elements
Compatibility with behavioral patterns	11.The space be in order in is details and generalities
Identity	12.The space design be in accordance with culture, customs and traditions of the region
Eventuality Collective memory	13.Cultural events occur in the space
Eventuality Collective memory	14.Social events occur in the space
Eventuality Collective memory	15.Religious events occur in the space
Eventuality Collective memory	16.Sporting events occur in the space
Compatibility with behavioral patterns	17.Peace be its distinct characteristic
Complexity	18.The space be full of soul and liveliness
liberty	19.There be no noting eye in the space
permeability	20.The space be of broad and wide perspective
liberty	21.There be freedom and discretion for women to do different activities in the space
permeability	22.The space be clear and open
Complexity	23.There be indistinct and discoverable points in the space
Attention to the climate	24.Existence of natural elements be highlighted (trees, fountains, birds and the likes
variety	25.Different social stratums attend the public space
permeability	26.The spaces be just near to our residence/house
Security	27.The space security be provided by some organs of government (such as police)
variety	28.Both genders (male and female) attend the space
Compatibility with behavioral patterns	29.Suites and all other welfare equipment be included
Complexity	30.The space be a noisy and lively place due to crowds of people
permeability	31.At least the space be located in urban texture
Identity	32.The space be walled
Compatibility with behavioral patterns	33.The space could be used at different hours of the day and in different seasons
Identity	34.The space be a warm and friendly place

**Table 4:** Determining the degree of importance of each environmental priority by the population using T-test

Environmental priorities	T-Test 1	T-Test 2	T-Test 3	T-Test 4	T-Test 5	T-Test 6	T-Test 7	T-Test 8	T-Test 9	T-Test 10
	(The degree of importance 1)	(The degree of importance 2)	(The degree of importance 3)	(The degree of importance 4)	(The degree of importance 5)	(The degree of importance 6)	(The degree of importance 7)	(The degree of importance 8)	(The degree of importance 9)	(The degree of importance 10)
Permeability	24.276	19.591	14.905	10.22	5.534	0.849	-13.208	-8.522	-13.208	-17.893
Security	6.508	-0.961	-8.431	-15.901	-23.37	-30.84	-53.249	-45.779	-53.249	-60.718
Compatibility with behavioral patterns	11–510	4.975	-1.559	-8.094	-14.628	-21.163	-40.766	-37.233	-4.0766	-47.301
Eventuality	21.286	16.175	11.064	5.953	0.842	-4.269	-19.601	-14.49	-19.601	-24.712
Attention to the climate	27.737	22.226	16.714	11.203	5.692	0.18	-16.354	-10.842	-16.354	-21.865
Collective memory	57.154	50.931	44.707	38.484	32.261	26.037	7.367	13.59	7.367	1.143
Liberty	40.024	34.256	28.488	22.72	16.952	11.184	-6.12	-0.352	-6.12	-11.887
Variety	39.676	34.267	28.859	23.45	18.042	12.633	8.224	1.816	-3.953	-9.001
Identity	27.327	22.228	17.128	12.029	6.929	11.83	-9.27	-8.369	-16.469	-18.905
Complexity	29.467	15.005	12.543	16.081	10.619	-12.843	-7.384	-11.766	-13.288	-20.69
The degree of importance	1	2	3	4	5	6	7	8	9	10
Factor	Security	Compatibility with behavioral patterns	Eventuality	Permeability	Attention to the climate	liberty	Collective memory	variety	Complexity	Identity

As mentioned above, the second purpose of the study is to examine the correlation between respondents’ individual characteristics (age, education, work status and marital status) and components related to their desire to be present in the urban space. Since the data are in the normal range, the correlation of the variables has been examined using the Pearson parametric correlation and Cramer's V correlation coefficient tests. Tables 5, 6, 7, 8 show the correlation between individual characteristics and components related to women’s presence over 18.

**Table 5:** The correlation between the education of the respondents and the components related to women’s presence in urban spaces (source: authors)

Personal factors	Permeability	Security	Compatibility with behavioral patterns	Eventuality	Attention to the climate	Complexity	variety	liberty	Identity	Collective memory	sum
Education Level	0.034	0.089	0.071	0.031	-0.079	0.081	0.081	0.589	0.021	0.036	0.148

**Table 6:** The correlation between the age of the respondents and the components related to women’s presence in urban spaces

Personal factors	Permeability	Security	Compatibility with behavioral patterns	Eventuality	Attention to the climate	Complexity	variety	liberty	Identity	Collective memory	sum
Age	-0.203	0.644	-0.206	-0.187	-0.253	0.097	-0.104	0.703	0.784	0.805	0.386

**Table 7:** The correlation between the marital status of the respondents and the components related to women’s presence in urban spaces

Personal factors	Permeability	Security	Compatibility with behavioral patterns	Eventuality	Attention to the climate	Complexity	variety	liberty	Identity	Collective memory	sum
Marital Status	0.201	0.443	0.367	0.338	0.031	0.021	0.05	0.402	0.216	0.011	0.209

**Table 8:** The correlation between the employment status of the respondents and the components related to women’s presence in urban spaces

Personal factors	Permeability	Security	Compatibility with behavioral patterns	Eventuality	Attention to the climate	Complexity	variety	liberty	Identity	Collective memory	sum
Work Status	0.107	0.485	0.08	0.553	0.202	0.012	0.218	0.317	0.014	0.011	0.179

First, it should be noted that the results of this section have not been studied in similar studies so far; so they can be considered as a research innovation. According to the results, among the individual characteristics of the respondents, the age component compared to other individual characteristics, had the highest correlation (average correlation of 0.386) with the components related to women’s presence in urban spaces. The component of collective memory with a correlation of 0.805, identity with a correlation of 0.784, liberty with a correlation of 0.703, and finally, safety and security with a correlation of 0.644 had a strong and positive correlation with the respondents’ age. In other words, the older the women responders, the more the mentioned components attracted their attention.

After that, marital status had a weak and positive correlation (0.209) with the components related to women’s presence in urban public spaces. This feature has a relatively higher correlation with safety and security (0.443) and liberty (0.402). Work status is in the third place in terms of correlation with the components affecting women’s presence in urban spaces (0.179). The components of eventuality (0.553), safety, and security (0.485) have a positive and moderate correlation with this feature; and the component of liberty is in the third place (0.317). It means that employed people compared to unemployed people have shown a greater tendency to consider these three components as influential factors in public presence. Finally, the education feature had the least correlation with the components related to the presence of individuals (0.148). However, this feature has a strong and positive correlation with the liberty component (0.589). This means that the higher the level of education of the respondents, the greater their tendency to consider this component as an influential factor in public presence.

## Discussion

Women are one of the groups whose need to be present in urban public spaces to establish normal social interactions has been ignored more than others. This is while continuous and dynamic presence of these social groups in urban public spaces guarantees sustainability of such spaces. The results of this study indicate that more than any other factors, promoting a sense of social security on the one hand and providing a safe and secure environment on the other hand, provide the ground for more women’s presence (over 18 years) in urban public spaces.

According to the questionnaire results using T-test, the environmental priorities of women over 18 in Shiraz for active presence in urban public spaces can be listed as follows, respectively: 1) safety and security, 2) compatibility with behavioral patterns, 3) eventuality, 4) permeability, 5) paying attention to climate, 6) liberty, 7) collective memory, 8) variety, 9) complexity, and 10) identity.

Among these, “security” has been mentioned as the most important priority in motivating women for active social participation in urban public spaces. Security is one of the basic rights and a pre-requirement for human welfare and peace. The feeling of security is not always directly related to real security; and its presence or absence does not necessarily mean enjoyment or deprivation of real security. It seems that providing objective and subjective security of women in urban spaces is a precondition for creation of a desirable and healthy urban public space. This feature is the first priority for the population of this study and confirms the results of a 2005 study by the Division for the Advancement of Women in France. This study stated that most of the rapes and violations of women’s rights, which cause objective and subjective insecurity, take place in urban public spaces. Also, the results of the present study on security being an environmental priority are consistent with the study by Gebremedhin (2022) [17] (which considers lack of security as a basis for limiting mobility of women in urban public spaces), Ghani et al. [18] (who believed that women’s tendency to walk is far more in neighborhoods with lower crime rates), Soltani & Ghanbari [59] (saying that the lack of security and comfort is the important factor affecting the absence of women in public spaces), Harvey, et al. [24] (saying that women consider urban spaces less secure from crime compared to men), Sadeghi, et al. [53] (who believed that the main reason for lower women’s presence in urban public spaces alone or at nights is their not feeling secure), Navarrete-Hernandez et al. [43] (saying that women Concern for personal safety thus has often been found to preclude women from full and meaningful inclusion in public spaces, thus limiting opportunities to effectively reap the benefits to one’s wellbeing that come from accessing the public realm), the study of lak et al. [35] (show that three main themes consisting of psychological, functional and environmental safety affect on elder women’s sense of safety. Psychological safety includes fears of falling, getting lost, social limitation, anxiety, and social support or capital, while functional safety consists of concerns about public transportation, walkability, and physical activity. Finally, environmental safety comprises of apprehension of road traffic accidence, criminals, upkeep, incivility, and nuisance).

Krenichyn [33] (saying that women are worried about their safety and perceived levels of danger in their neighborhoods such as crime rate, availability of safe places for doing sports, and feeling secure when walking during the day), as well as results of the studies by Soraganvi [60], Borumand & Rezaee [9], Arjmand [3], and Durmisevic & Sariyildiz (2001) [12].

The environmental priority of “compatibility with behavioral patterns” being the second important component is consistent with the 2018 study by Rayatidamavandi, Faizi & Mozaffar in [50] (who consider the existence of green spaces, quality of sidewalks and passages, and the design of public spaces as factors affecting women’s presence in urban parks), the study by Askarizad & Safari [4] (stating that social interactions have a profound effect on women’s behavior in urban spaces such that this behavioral effect, which arises from the quality of the built environment, is transmitted to individuals and affects their behavior in their personal lives), the study by Jin & Whitson [28] (who believe while women have access to a variety of public leisure spaces, they are more willing to be present in the spaces where are not inherently masculine in their view such as cafes and shopping malls), as well as the studies by Khalili & Nayyeri Fallah [31], Van Hagen & Bron [67] and Matsuoka & Kaplan [40].

The environmental priority of “eventuality” being in the third place from the viewpoint of the population confirms the results by Borumand & Rezaee [9]. Borumand & Rezaee introduce urban space performance in terms of scale of activities and times of space activity as an effective factor in promoting gender equity in urban parks, which confirms results of the present study. The present study is also consistent with the study by Fine [16] (who believes that eventual experiences are a source of social mobility in men and women), and Kern (2016) [30] (who states that women use the events in their neighborhoods or urban spaces to present themselves as members of the community).

According to the results of this study, the environmental priority of “permeability” is in the fourth place of importance for women’s presence. This is consistent with the study by Gebremedhin (2022) [17] that emphasizes limitations of women’s mobility in urban public spaces and believes that women’s patterns of movement and behavior in urban spaces are affected by fear and limitations of access to different spaces. Permeability component in the sense of creating access to opportunities for citizens to participate in urban spaces also confirms the results of the studies by Khalili & Nayyeri Fallah in [31], Soltani & Ghanbari in [59] and Alamdari & Habib in [1]. Also, results of the present study on environmental priority of permeability are consistent with the study by Beebeejaun (2017) [7] (who states that urban planning plays a vital role in supporting women in having access to urban spaces) and also the studies by Weber [69], Purcell (2003) [48].

The importance of the environmental priority of “paying attention to climate”, as the fifth component in the sense of providing climatic comfort and favorable temperature conditions is consistent with the studies by Khalili & Nayyeri Fallah [31] and Alamdari & Habib [1], both of which consider climatic comfort as one of the most important factors in encouraging women’s presence in public spaces, Williams, et al. (2019) (who considered climate change to be effective in people’s use of urban space and considers innovative and integrated approaches to development and environmental sustainability at the local level to be necessary), Schofield & Gubbels [56] (who believed that women due to gender inequities are particularly vulnerable to climate changes), Amindeldar, et al. [2] (who concluded that under cold winter conditions, the comfort zone for women seems to be smaller, indicating that women are more sensitive to adverse winter conditions), as well as the studies by Thorsson et al. (2007) [66], Thorsson et al. [65] and Eliasson [13].

The “liberty” component, in the sense of possibility of performing various social activities by all citizens in urban spaces, being in the sixth place, confirms the studies by Borumand & Rezaee [9]; Sunikka-Blank, et al. [63] (stating that lack of favorable social spaces reduces women’s potential to use social capital through collective activities); Gholamhosseini, et al. [19] (who considered the flexible meanings of public spaces and creation of a sense of place to be effective in women’s perceptions and experiences of public spaces and therefore public spaces must meet certain cultural and environmental criteria for women), and Sunaryo et al. [62] (stating that public space is an open space and is visually and physically accessible to all without exceptions where all members of society have the right to freely choose activities and people do various political, economic, and cultural activities and social interactions in a common environment).

Existence of “collective memory” in a space, ranked seventh, makes it possible to recall shared memories between individuals. The related results are consistent with the studies by Lak & Hakimian [34] (believing that collective memory, which consists of objective and subjective dimensions including place, events, activities, history, and values links people under a common identity and is usually kept alive through group interactions and reminding focused on collective memory in place), and Bagheri (2014) [5] (saying that some women walk around traditional architecture and urban spaces to enjoy the sense of place and remember the good old days).

The eighth place for “variety” component is also consistent with the studies by Krenichyn [33] (believing that the variety of uses and users in public spaces is the main focus of women), Hataminejad, et al. [25] (stating that variety and attractiveness of the environment including variety in the layout, facade, and body of buildings, variety in provision of commercial and leisure services such as retails, restaurants and cafeterias, and presence of peddlers and traveling artists in the environment have the greatest effect on women’s presence in urban spaces), Mohammadi & Rafiee [41] (stating that variety is one of the indicators that increases women’s presence in the neighborhood), Raymond, et al. [51,14], and Matsuoka & Kaplan [40].

The “complexity” of space was another variable studied in this study, which was ranked ninth. Increased complexity means visual richness. Results of the present study on environmental priority of diversity are consistent with the studies by Khalili & Nayyeri Fallah [31] (stating that women’s public life is not limited to a single space, but is shaped in a multifunctional spatial complex with a combination of commercial, social, and religious situations), Portella [47] (arguing that the principle of complexity, which is related to Gestalt laws, affects user’s perception and evaluation of the built environment), Maclean [39], and Ewing & Clemente [15].

Finally, “identity” and sense of belonging to a space was the last criterion for women’s presence in urban space in this study. In 2004, Ortiz et al. [45] examined the relationship between sense of place and identity and women’s presence in Barcelona’s urban spaces. According to this study, identity of space leads to women’s more willingness to use public facilities in space and ultimately increases their motivation to be present in the space. Results of the present study on environmental priority of identity, are consistent with the study by Golkowska [20] (stating that in spaces with identity we can see significant increase in women’s presence and visibility in public spheres, especially in educational, employment, and sports spaces), Khan (2007) [32] (who believes that homogeneous neighborhoods having residents with identical identities have a greater understanding of the security resulting from “being with one’s own congeners”), Jabareen [27], Secor [57], Williams & Vaske [70], and Vaske & Kobrin [68].

## Conclusion

Urban space is an organized phenomenon that provides the basis for the formation of society and social relations and therefore has always been considered and used by the general public. Women make up about half of the world’s population. Although their equal right to be present in urban spaces has always been emphasized, in some cases they have encountered deterrents and have been deprived of the right to be present in urban spaces. The present study has examined the indicators related to women’s presence in urban spaces in the city of Shiraz. In today’s world, creating a balanced society is not possible without participation of women, and this increases the importance of addressing strategies to improve women’s participation in urban spaces. The present study was an attempt to extract the indicators related to women’s presence in urban spaces and to rank them based on their priorities from the viewpoint of citizens. According to the results, the feeling of security was identified as the most important factor associated with women’s presence. Security is an important phenomenon that is a necessity for individuals and societies, and lack of it leads to dangerous consequences because security is an emotional and perceptual phenomenon and often involving the psychological feeling of citizens about threatening factors. Since the type of feeling of crime is different among men and women, the type of their feeling of security in urban spaces is different. Even in spaces where men are more exposed to violence, women feel more insecure and afraid. In general, the feeling of insecurity is a threatening factor for the presence of people, especially women, in urban spaces. It should be noted that lack of a sense of social security in public spaces, leads to withdrawal of people, especially more vulnerable groups such as women, from social and urban life, distrust of others, avoidance of certain places, and in general, reduced tendency to attend and continue voluntary and social activities in urban spaces. Meanwhile, crime prevention approach as the most important approach to prevent crime and create environmental security is considered by designers, which means a set of environmental design principles that affect crime prevention and promoting environmental security. Designing secure public spaces for women is creating spaces that increase the feeling of security and reduce the features that intensify the feeling of insecurity in women. Thus, shaping secure spaces is always one of the important priorities of urban space designers. With a special focus on identifying factors such as lighting, furniture, space monitoring, the type and amount of vehicles and pedestrians referring to the space, crime spots, etc. in urban spaces they look for ways to increase security of all citizens by exploiting the potential of public spaces.

In addition to security, other indicators related to women’s presence in urban spaces have also been mentioned including compatibility with behavioral patterns, eventuality, permeability, paying attention to climate, liberty, collective memory, variety, complexity, and identity. Addressing strategies to increase women’s presence in urban spaces by considering each of these indicators is as an effort to provide justice and equity and remove limiting barriers including physical concepts, and social and cultural conditions, etc. in urban spaces. Paying attention to these issues in creating and maintaining urban space can be effective in improving the efficiency of such spaces and avoiding creation of an urban space that leads to ignoring some people. Urban design can meet the needs of citizens by creating a suitable environment. Utilizing the principles of urban design with a specific goal is effective in creating a space that is appropriate to the wishes of people. In today's world, creating urban spaces allowing the presence of all people of different age and gender groups is one of the main goals of urban design.

Creating such balanced spaces requires the full attention of designers and city managers. The selection of urban spaces based on the type of performance in future studies can complement the research results. Segregation of the studied spaces in terms of the type of space (such as public and semi-public spaces) as well as their dominant function and activity (such as commercial, recreational, etc.) and providing indicators affecting the presence of women in each of urban public spaces can help increase the accuracy of research results. In addition, the segregation of women who use space in terms of age, type of activity, presence in public or alone in space, etc. is another important factor that can be considered in future research.

## Supplementary Information

**Additional file 1: **Questionnaire.

## Data Availability

The datasets used and/or analysed during the current study available from the corresponding author on reasonable request.
