# Supplementary material for: Women in a safe and healthy urban environment: environmental top priorities for the women’s presence in urban public spaces
Source: BMC Womens Health. 2023 Apr 6;23:163. doi: 10.1186/s12905-023-02281-8 (PMC10077683; doi:10.1186/s12905-023-02281-8)
Supplement: Supplementary file 1 — Additional file 1: Questionnaire. [file 12905_2023_2281_MOESM1_ESM.doc]

**Questionnaire**

In order to promote the quality of public urban spaces, and make them commensurate with Moslem women's criteria, I am to collect some pieces of information. Your cooperation as female citizens provide provide grounds for development and promotion of the spaces in question. Would you be kind enough to answer the following questions? Thank you in advance for your cooperation and help.

Education:

Place of residence:

Age:

- In your view, what are the very major characteristics and criteria of a desirable public urban space to attend? (Please prioritize the following criteria according to their importance)

| **Characteristics and Criteria for Attendance** | **Prioritization** |
| --- | --- |
| Permeability  Security  Variety  Attention to the Climate  Complexity  Identity  Liberty  Eventuality  Compatibility with Behavioral Patterns Collective Memory |  |

Please mention any other item which may be of importance to you other than the above-mentioned.

Please check your answers to the following questions

| **Strongly disagree** | **disagree** | **No opinion** | **agree** | **Strongly agree** | **Question** |
| --- | --- | --- | --- | --- | --- |
|  |  |  |  |  | 1. The space be of mental health atmosphere |
|  |  |  |  |  | 1. People of different ages attend that space |
|  |  |  |  |  | 1. View, paint, and materials used in the space be in harmony with the surrounding environment |
|  |  |  |  |  | 1. The place being far or near is of no importance |
|  |  |  |  |  | 1. The public space be clean and far from sound and air pollution |
|  |  |  |  |  | 1. The space be defensible (little crime and malefaction be committed in the space) |
|  |  |  |  |  | 1. Public access to the public urban space be considered. |
|  |  |  |  |  | 1. Diverse uses and performance be available in the space |
|  |  |  |  |  | 1. Creative and diverse color composition be used in the space. |
|  |  |  |  |  | 1. - There be integrity in the whole space and little dispersion of elements. |
|  |  |  |  |  | 1. The space be in order in is details and generalities |
|  |  |  |  |  | 1. The space design be in accordance with culture, customs, and traditions of the region. |
|  |  |  |  |  | 1. Cultural events occur in the space. |
|  |  |  |  |  | 1. Social events occur in the space. |
|  |  |  |  |  | 1. Religious events occur in the space. |
|  |  |  |  |  | 1. Sporting events occur in the space. |
|  |  |  |  |  | 1. Peace be its distinct characteristic. |
|  |  |  |  |  | 1. The space be full of soul and liveliness. |
|  |  |  |  |  | 1. There be no noting eye in the space. |
|  |  |  |  |  | 1. The space be of broad and wide perspective. |
|  |  |  |  |  | 1. There be freedom and discretion for women to do different activities in the space. |
|  |  |  |  |  | 1. The space be clear and open |
|  |  |  |  |  | 1. There be indistinct and discoverable points in the space. |
|  |  |  |  |  | 1. Existence of natural elements be highlighted (trees, fountains, birds and the likes. |
|  |  |  |  |  | 1. Different social stratums attend the public space |
|  |  |  |  |  | 1. The spaces be just near to our residence/house. |
|  |  |  |  |  | 1. The space security be provided by some organs of government (such as police) |
|  |  |  |  |  | 1. Both genders (male and female) attend the space. |
|  |  |  |  |  | 1. Suites and all other welfare equipment be included. |
|  |  |  |  |  | 1. The space be a noisy and lively place due to crowds of people. |
|  |  |  |  |  | 1. At least the space be located in urban texture |
|  |  |  |  |  | 1. The space be walled. |
|  |  |  |  |  | 1. The space could be used at different hours of the day and in different seasons. |
|  |  |  |  |  | 1. The space be a warm and friendly place. |
